# Acidic phospholipids govern the enhanced activation of IgG-B cell receptor

**DOI:** 10.1038/ncomms9552

**Published:** 2015-10-06

**Authors:** Xiangjun Chen, Weiling Pan, Yinqiang Sui, Hua Li, Xiaoshan Shi, Xingdong Guo, Hai Qi, Chenqi Xu, Wanli Liu

**Affiliations:** 1 MOE Key Laboratory of Protein Sciences, Collaborative Innovation Center for Diagnosis and Treatment of Infectious Diseases, School of Life Sciences, Tsinghua University, Beijing 100084, China; 2 State Key Laboratory of Molecular Biology, National Center for Protein Science Shanghai, Institute of Biochemistry and Cell Biology, Shanghai Institutes for Biological Sciences, Chinese Academy of Sciences, Shanghai 200031, China; 3 Tsinghua-Peking Center for Life Sciences, Laboratory of Dynamic Immunobiology, School of Medicine, Tsinghua University, Beijing 100084, China; 4 School of Life Science and Technology, ShanghaiTech University, Shanghai 200031, China

## Abstract

B cells that express the isotype-switched IgG-B cell receptor (IgG-BCR) are one of the driving forces for antibody memory. To allow for a rapid memory IgG antibody response, IgG-BCR evolved into a highly effective signalling machine. Here, we report that the positively charged cytoplasmic domain of mIgG (mIgG-tail) specifically interacts with negatively charged acidic phospholipids. The key immunoglobulin tail tyrosine (ITT) in mIgG-tail is thus sequestered in the membrane hydrophobic core in quiescent B cells. Pre-disruption of such interaction leads to excessive recruitment of BCRs and inflated BCR signalling upon antigen stimulation, resulting in hyperproliferation of primary B cells. Physiologically, membrane-sequestered mIgG-tail can be released by antigen engagement or Ca^2+^ mobilization in the initiation of B cell activation. Our studies suggest a novel regulatory mechanism for how dynamic association of mIgG-tail with acidic phospholipids governs the enhanced activation of IgG-BCR.

## Abstract (web summary)

Adaptive immunity is the memory of previously experienced pathogens, where B cells establish a rapid antibody response upon IgG-B cell receptor activation. Here, Chen *et al*. show that sequestration of the cytoplasmic domain of mIgG by plasma membrane phospholipids prevents inappropriate activation of IgG-BCR signalling.

## Graphical abstract

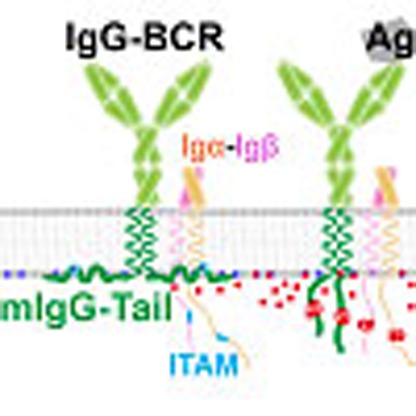

A key feature of the adaptive immunity is the memory for previously exposed pathogens^1^. Antibody memory is a major component of memory immunity and provides the basis for nearly all currently used human vaccines. Upon the first encounter with an antigen, the IgM- and IgD-B cell receptor (BCR) expressing naive B cells generate slow and low-titred primary antibody responses^2^. Memory B cell that expresses class-switched IgG-BCR is one of the driving forces responsible for IgG antibody memory, leading to rapid and high-titred IgG antibody responses upon antigen recall. Both naive and memory B cells utilize the surface BCRs to recognize antigens and initiate signalling^3^. The BCR molecule is a complex composed of a membrane-bound immunoglobulin (mIg) and a heterodimer of Igα and Igβ^4^,^5^. It is generally accepted that the function of the mIg is to recognize antigens, while the Igα and Igβ heterodimer initiates signalling through the immunoreceptor tyrosine activation motifs (ITAMs) in the cytoplasmic domains^6^. The cytoplasmic domains of mIgM and mIgD contain only three amino acid (aa) residues, KVK, and thus cannot trigger signalling. In contrast, all mIgG subtypes harbour 28 aa cytoplasmic tails, which are highly conserved across species and contain an Immunoglobulin tail tyrosine (ITT) motif^7^,^8^,^9^.

Previous studies have demonstrated that the cytoplasmic domain of the mIgG (mIgG-tail) is both necessary and sufficient to confer the burst-enhanced activation of IgG-BCR expressing memory B cells and the subsequent memory IgG antibody responses^7^,^10^,^11^,^12^,^13^,^14^,^15^,^16^,^17^. Mechanistically, the conserved ITT motif in the mIgG-tail is phosphorylated upon antigen stimulation, which is followed by recruitment of growth-factor receptor-bound protein-2 (Grb2)^17^. Grb2 and its constitutively associated signalling molecule Bruton's tyrosine kinase (Btk) dramatically lower the activation threshold of 1-phosphatidylinositol-4,5-bisphosphate phosphodiesterase gamma-2 (PLCγ2) to potently amplify Ca^2+^ mobilization during IgG-BCR signaling^16^. Additionally, IgG-BCRs exhibit a dramatically enhanced capability to oligomerize and form microclusters in response to membrane-bound antigens^7^,^15^. All these studies improve our understanding of how IgG-BCR acquires burst-enhanced signalling via its conserved ITT motif within mIgG-tail. However, an equally important but understudied question is how IgG-BCR appropriately ensures an ordered signalling hierarchy of utilizing ITT signalling to amplify ITAM signalling in response to antigen stimulation.

Here, we systematically address this question by investigating whether membrane lipids can govern the potent signalling of the mIgG-tail. Recent studies highlight the sophisticated functional roles of acidic phospholipids in regulating membrane protein structure and function^18^,^19^,^20^,^22^. In this report, we use a combination of biochemical, biophysical and live-cell imaging approaches, and find that the positively charged mIgG-tail associated with the negatively charged acidic phospholipids in the inner leaflet of the plasma membrane (PM). The ionic protein-lipid interactions efficiently sequester the key ITT motif within the membrane hydrophobic core in quiescent B cells. Dynamic exposure of the ITT motif is induced by either antigen engagement or Ca^2+^ mobilization in activated B cells. The switch from a membrane-sequestered ITT motif in quiescent cells to a solvent-exposed ITT motif in activated cells ensures an ordered signalling hierarchy in the initiation of IgG-BCR activation. This concept is supported by the observation that IgG-BCR with a solvent-exposed mIgG-tail mutant (mIgG-Linker25-tail) exhibits an excessive recruitment of prominent BCR signalling microclusters into the B cell immunological synapse and more aggressive downstream signalling including inflated Ca^2+^ mobilization upon antigen stimulation, which ultimately lead to hyper-proliferation of B cells compared with the wild-type (WT) IgG-BCR. We also examined the dissociation mechanism of the mIgG-tail from the PM in activated B cells. Thus, we conclude that the evolutionarily conserved mIgG-tail is a potent signalling unit that can be governed by acidic phospholipids for an ordered and strict signalling hierarchy.

## Results

### mIgG-tail interacts with acidic but not zwitterionic lipids

**Figure 1:**
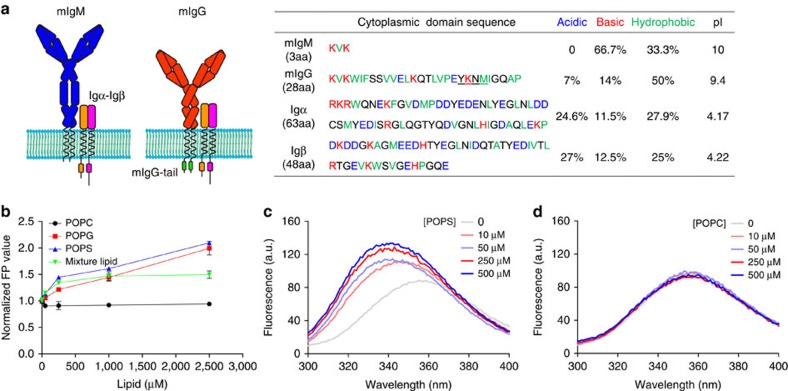
The cytoplasmic domain of mIgG interacts with acidic but not zwitterionic lipids. (**a**) Schematic representations of IgM-BCR, IgG-BCR and biochemical characteristic analysis of the cytoplasmic domain of mIgM, mIgG, Igα and Igβ. Acidic, basic and hydrophobic residues are respectively coloured in blue, red and green. ITT motif in mIgG-tail is underlined. (**b**) FP assay to measure binding of CP488-mIgG-tail peptide to zwitterionic lipid POPC bicelles (*q*=0.8), acidic lipid POPG or POPS bicelles and mixture lipid bicelles (60% POPC, 30% POPS, 10% POPG). FP value was detected in different lipids concentration of 10, 5, 250, 1 and 2.5 mM. All the FP value was normalized to FP value in solution. Bars represent mean±s.d. from three repeated experiments. (**c**,**d**) Tryptophan fluorescence emission spectrum assay to detect binding of mIgG-tail peptide to acidic lipid POPS bicelles (**c**) and zwitterionic lipid POPC bicelles (**d**) at different concentration of lipids as indicated. Shown was one representative of three independent experiments.

First, we analysed the biochemical characteristics of the cytoplasmic domain of each component of the BCR complex: Igα, Igβ, mIgG and mIgM (Fig. 1a). The cytoplasmic domains of Igα and Igβ have more acidic residues than basic residues and ∼25% hydrophobic residues, with pI values of 4.17 and 4.22, respectively (Fig. 1a). In contrast, the conserved mIgG-tail contains many basic and hydrophobic residues with a high pI value of 9.4, suggesting that the mIgG-tail might interact with the negatively charged inner leaflet of the PM. To address this hypothesis, we synthesized a peptide representing the mIgG-tail linked to an N-terminal CP488 dye. We first used a fluorescence polarization (FP) assay to detect the FP value of the CP488-mIgG-tail, which would dramatically increase upon membrane binding. In the context of large acidic lipid bicelles (*q*=0.8) composed of acidic 1-palmitoyl-2-oleoyl-sn-glycero-3-phosphoglycerol (POPG) or 1-palmitoyl-2-oleoyl-sn-glycero-3-phospho-L-serine (POPS), the FP value of the CP488-mIgG-tail progressively increased as the lipid concentration increased (Fig. 1b). In contrast, the FP value of the CP488-mIgG-tail did not change in the presence of zwitterionic 1-palmitoyl-2-oleoyl-sn-glycero-3-phosphocholine (POPC) bicelles (Fig. 1b). These results suggested that the mIgG-tail can specifically interact with acidic lipids but not with zwitterionic lipids. Next, we constructed a mixture lipid bicelle composed of 60% POPC, 30% POPS and 10% POPG to mimic the physiological lipid environment of the inner leaflet of the PM^23^. Similarly, we observed that as the concentration of the mixture bicelles increased, the FP value of the CP488-mIgG-tail continuously increased (Fig. 1b).

To exclude potential non-specific effects of the CP488 dye in these observations, we also synthesized a mIgG-tail peptide lacking CP488 and used a tryptophan fluorescence emission spectrum (TFES) assay to validate the specific interaction. We found that the tryptophan spectrum of the mIgG-tail showed an obvious blue shift and increased emission intensity in the presence of POPS bicelles, but not in the presence of POPC bicelles (Fig. 1c,d). This result indicated that the tryptophan residue changed its microenvironment when interacting with the acidic lipid bicelles. Taken together, these results demonstrate the specific interaction between cytoplasmic domain of mIgG and acidic lipids.

### mIgG-tail associates with the PM in quiescent B cells

**Figure 2:**
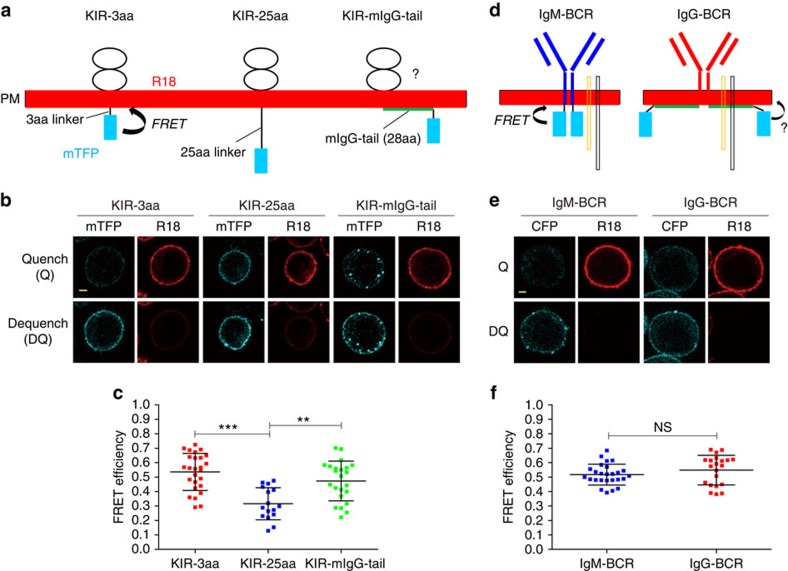
The cytoplasmic domain of mIgG associates with the PM in quiescent B cells. (**a**) Schematic representation showing the FRET system that was used to detect the interaction between mTFP (FRET donor) fused to C terminal of cytoplasmic tail and R18 dye (FRET acceptor) stained on the PM. FRET efficiency was calculated as detailed in the Method section. (**b**,**c**) Dequencing FRET to measure the FRET efficiency between mTFP and R18 in KIR-3aa, KIR-25aa and KIR-mIgG-tail expressing A20 B cells. FRET efficiency was calculated as detailed in the Method section. Representative confocal images were shown for each group (**b**). FRET efficiency was measured and plotted (**c**). Scale bar is 1.6 μm. (**d**) Similar as in **a** shown is a schematic FRET system to measure the interaction of the mIgG-tail with the inner leaflet of the PM in the context of IgG-BCR complex. Used as a control is mIgM-tail in the context of IgM-BCR complex. (**e**,**f**) Representative confocal images of dequenching FRET in IgM-BCR or IgG-BCR expressing J558L cells (**e**). FRET efficiency was calculated and compared in the context of IgM-BCR versus IgG-BCR (**f**). Scale bar, 1.6 μm. For all the FRET efficiency measurements, each dot represents one cell analysed in three independent experiments. Bars indicate mean±s.d. Two-tailed *t*-tests were used for the statistical comparisons. ***P<0.001; **P<0.01; NS, not significant.

We utilized a fluorescence resonance energy transfer (FRET) assay to examine whether the specific binding of the mIgG-tail to acidic lipid bicelles *in vitro* confers its interaction with the inner leaflets of the PM in quiescent B cells *in vivo*. We fused monomeric teal fluorescent protein (mTFP) as a FRET donor, to the C-terminus of the mIgG-tail (mIgG-tail-mTFP) and used the fluorescent membrane dye, octadecyl rhodamine B (R18) as a FRET acceptor, to stain the PM (Fig. 2a) following our published protocol^24^,^25^. Since the mIgG-tail has 28 aa residues with a fully extended length of ∼10 nm, we expected that high FRET efficiency between the mIgG-tail-mTFP and R18 should only be detected when the mIgG-tail associates with the inner leaflet of the PM.

First, we generated a chimera construct, designated KIR-mIgG-tail-mTFP, with the extracellular and transmembrane domains of an irrelevant NK cell receptor KIR2DL3 and the mIgG-tail-mTFP sequence as the cytoplasmic domain. We also set up two standard constructs with 3aa or 25aa flexible linker between the transmembrane domain of KIR2DL3 and mTFP, designated KIR-3aa-mTFP and KIR-25aa-mTFP, respectively^24^,^25^. KIR-3aa-mTFP served as the high FRET efficiency control and KIR-25aa-mTFP as the low FRET efficiency control (Fig. 2a). We acquired the dequenching FRET efficiency by measuring the mTFP fluorescence intensity change after photo-bleaching of the acceptor R18 dye. Using this FRET system, we measured the FRET efficiency of KIR-3aa-mTFP, KIR-25aa-mTFP and KIR-mIgG-tail-mTFP in A20 B cells. We found that the KIR-mIgG-tail-mTFP had a high FRET efficiency of ∼50%, which was comparable to the FRET efficiency observed for the KIR-3aa-mTFP (Fig. 2b,c). Both of these FRET efficiencies were significantly higher than that of the KIR-25aa-mTFP (∼29%) (Fig. 2b,c). These FRET data suggest that the mIgG-tail in the chimera construct associates with the PM in quiescent B cells.

Next, we assessed the potential interaction of the mIgG-tail with the inner leaflet of the PM in the IgG-BCR complex. We used IgM-BCR as a positive control since the cytoplasmic tail of the mIgM contains only three aa residues, KVK (Fig. 2d). IgG-BCRs exhibited a high FRET efficiency that was comparable to IgM-BCRs (Fig. 2e,f), indicating that the mIgG-tail in the context of the IgG-BCR associated with the PM in quiescent B cells.

### Lipid binding sequesters ITT motif within the PM

**Figure 3:**
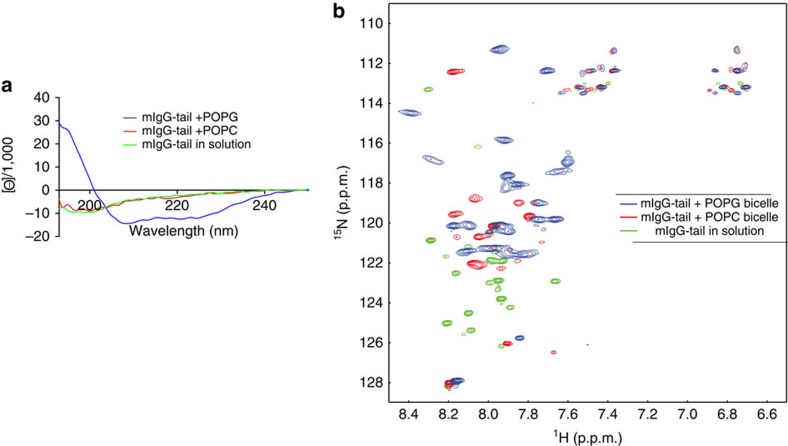
Membrane binding induces partial helical folding of mIgG-tail. (**a**) Far UV CD spectra of mIgG-tail (20 μM) in solution or in the presence of POPG or POPC bicelles (0.5 mM). (**b**) Superimposed ^15^N–^1^H TROSY spectra of mIgG-tail in phosphate buffer solution (pH 6.7) (green) and in the presence of POPG/DHPC (blue) or POPC/DHPC bicelles (red) (q=0.8).

Additionally, we assessed whether lipid binding alters the conformation of the mIgG-tail. Big lipid bicelles were used to provide physiologically relevant membrane environment^26^. We found that the far UV circular dichroism (CD) spectrum of the mIgG-tail in solution was characteristic of an unstructured protein (Fig. 3a). However, in the presence of acidic POPG, the mIgG-tail exhibited an obvious partial helical folding. In contrast, no secondary structural change was observed when zwitterionic POPC bicelles were added to the mIgG-tail solution (Fig. 3a). These results indicate that acidic, but not zwitterionic phospholipids induce lipid-dependent α-helical folding of the mIgG-tail.

Furthermore, we used multidimensional nuclear magnetic resonance (NMR) spectroscopy to study the conformation of the membrane-bound mIgG-tail in the context of POPG bicelles and the solvent-exposed mIgG-tail in solution. We performed 2D ^15^N–^1^H transverse relaxation-optimized spectroscopy (TROSY) with ^15^N-labelled mIgG-tail in solution, with POPG bicelles or with POPC bicelles. The CD experiments in Fig. 3a reflect the secondary structure of the protein, whereas the ^15^N-TROSY spectra in Fig. 3b reflect the chemical environment of each residue. The TROSY spectrum of the mIgG-tail in solution showed sharp signals with narrow distribution, indicating that the mIgG-tail in solution was highly flexible and unstructured. In contrast, in the presence of POPG bicelles, the spectrum was fully different, which indicated that binding to POPG changed the chemical environment of mIgG-tail (Fig. 3b). Moreover, the spectrum of mIgG-tail+POPG sample showed good signal dispersion and homogeneous signal intensities, indicating mIgG-tail should be well folded in the context of POPG (Fig. 3b). The spectrum of mIgG-tail in the zwitterionic POPC bicelles was different from those of the other two samples (Fig. 3b). In general, its signal dispersion was narrow and the signal intensities of different residues were inhomogeneous. These NMR data suggested that there might be weak and non-specific hydrophobic interaction between mIgG-tail and POPC that could cause mild chemical environment change but it could not induce the conformational change of mIgG-tail. This conclusion is consistent with the abovementioned FP and TFES data.

We tested whether the key signalling tyrosine (Y21) located in the ITT motif was sequestered within the membrane hydrophobic core. We carried out two-dimensional (2D) aromatic-filter nuclear overhauser effect (NOE) experiments to measure specific NOE signals (distance <5 Å) between aromatic protons and the lipid protons. NOE experiments were performed with ^13^C, ^15^N-labelled mIgG-tail with POPG bicelles. Y21 had substantial NOE signals to lipid acyl chains (Supplementary Fig. 1), which indicated that the tyrosine side chain was inserted into the membrane hydrophobic core. Moreover, W5 and F7 at the N-terminus of the mIgG-tail also had NOE signals to lipid acyl chains (Supplementary Fig. 1), implying that the entire mIgG-tail was bound to the PM with Y21 buried in the membrane hydrophobic core.

### Basic residues account for mIgG-tail and PM interaction

**Figure 4:**
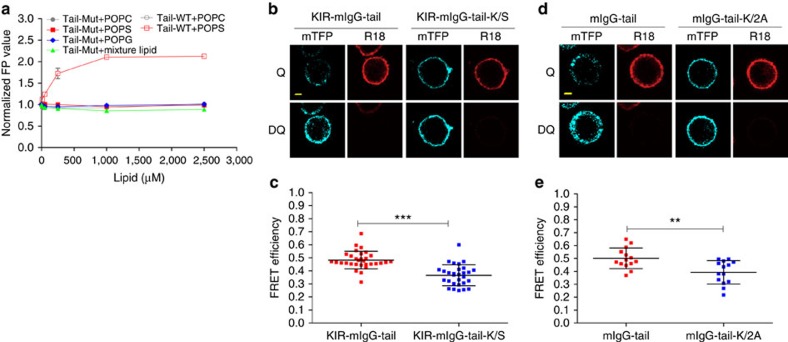
Basic residues in mIgG-tail are critical for the association with the PM. (**a**) FP assay to measure the binding of CP488-mIgG-tail-K/S peptide (tail-Mut) and CP488-mIgG-tail peptide (tail-WT) to lipid POPS, POPG or POPC bicelles and mixture lipids bicelles. Different lipids concentration of 10, 50, 250 and 2.5 mM were used in the measurement. Bars represent mean±s.d. from three repeated experiments. (**b**,**c**) Representative images of dequenching FRET in KIR-mIgG-tail or KIR-mIgG-tail-K/S expressing A20 B cells (**b**). FRET efficiency of mIgG-tail and mIgG-tail-K/S were detected and compared as described above (**c**). Scale bar, 1.6 μm. (**d**,**e**) Dequenching FRET was performed and FRET efficiency was statistically analysed in Ramos B cells expressing IgG-BCR with either mIgG-tail or mIgG-tail-K/2A. Each dot represents one cell analysed in three independent experiments. Bars indicate mean±s.d. Two-tailed *t*-tests were used for the statistical comparisons. ***P<0.001; **P<0.01.

Considering that the mIgG-tail specifically binds to acidic lipids, we tested whether the basic residues play dominant roles in the interaction of the mIgG-tail with the PM. We first synthesized a mutant peptide CP488-mIgG-tail-K/S (referred to as tail-Mut in Fig. 4a) with all the basic lysine (K) resides replaced with non-charged serine (S) residues. Compared to the WT CP488-mIgG-tail peptide (referred to as tail-WT in Fig. 4a), CP488-mIgG-tail-K/S lost the ability to interact with acidic lipids when examined in a FP assay (Fig. 4a). Additionally, a FRET analysis showed that the KIR-mIgG-tail-K/S exhibited significantly decreased FRET efficiency compared with that of the WT KIR-mIgG-tail (Fig. 4b,c). These data suggest that the mIgG-tail-K/S mutant does not associate with the PM compared with the WT mIgG-tail.

Next, we attempted to confirm this conclusion in the context of the IgG-BCR complex. The KVK sequence at the membrane-proximal region of the mIgG-tail and the mIgM-tail is known to be important in maintaining the function and membrane localization of BCRs^27^. Thus, we chose to only mutate the two basic residues at the membrane distal region of the mIgG-tail (mIgG-tail-K/2A). Compared to the WT mIgG-tail, the mutant mIgG-tail-K/2A in the context of the IgG-BCR exhibited a significantly decreased capability to associate with the PM (Fig. 4d,e). Thus, these results demonstrate that the basic residues in the mIgG-tail are critical for the association with the PM.

### Design of a solvent-exposed mutant mIgG-Linker25-tail

**Figure 5:**
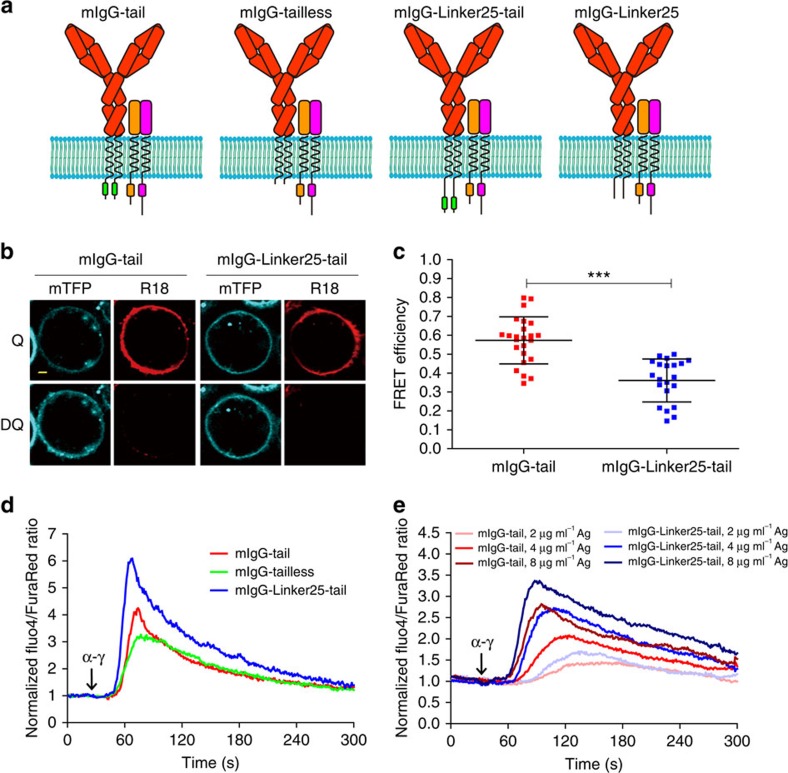
Solvent-exposed mIgG-tail triggers inflated Ca^2+^ mobilization. (**a**) Schematic representations of IgG-BCR with WT or three mutant forms of mIgG-tail. The aa sequence of the cytoplasmic tail of each mIgG is: mIgG-tail (KVK-WIFSSVVELKQTLVPEYKNMIGQAP); mIgG-tailless (KVK), mIgG-Linker25-tail (KVK-GSGSGPGSPSGPGSPSGPSGPGSGH-KVKWIFSSVVELKQTLVPEYKNMIGQAP), mIgG-Linker25 (KVK-GSGSGPGSPSGPGSPSGPSGPGSGH). The ITT signalling motif on mIgG-tail is depicted in green colour. The ITAM signalling motifs on Igα and Igβ are also depicted. (**b**,**c**) FRET assay in Ramos cells expressing mIgG-Linker25-tail to confirm the solvent-exposed state of mIgG-tail in quiescent cells. Scale bar is 1.6 μm. Each dot represents one cell analysed in three independent experiments. Bars indicate mean±s.d. Two-tailed *t*-tests were used for the statistical comparisons. ***P<0.001. (**d**) Ca^2+^ mobilization analysis by Flow cytometry in Ramos B cells expressing mIgG-tail, mIgG-tailless or mIgG-Linker25-tail. Arrow indicates time point of the anti-mouse IgG (*α-γ*) stimulation. One representative data from three independent experiments is shown. (**e**) Ca^2+^ mobilization analysis of mIgG-tail and mIgG-Linker25-tail in Ramos B cells after different concentration of *α-γ* stimulation.

Next, we attempted to evaluate the function of IgG-BCR with a solvent-exposed cytoplasmic tail in comparison to the WT IgG-BCR with a membrane-sequestered cytoplasmic tail. The aforementioned mIgG-tail-K/2A mutant unfortunately cannot be used for these functional experiments because we consistently observed that IgG-BCRs with mIgG-tail-K/2A showed significantly impaired cell surface expression compared with the case of mIgG-tail (Supplementary Fig. 2a), which excluded the feasibility of accurately comparing the activation of B cells expressing mIgG-tail-K/2A versus B cells expressing mIgG-tail. The significantly reduced surface expression was also reported in T-cell receptor (TCR) studies showing that the mutation of key basic residues within the cytoplasmic domain of the CD3ɛ dramatically impaired the expression of TCR on the PM^28^,^29^. Therefore, to generate a solvent-exposed mIgG-tail, we cannot modify the native sequence of the mIgG-tail in which the basic residues are in fact highly conserved across species (Supplementary Fig. 3). Instead we chose the strategy of inserting a 25 aa flexible linker between the transmembrane domain of the mIgG and N-terminus of the mIgG-tail (mIgG-Linker25-tail) (Fig. 5a). We chose the 25 aa flexible linker based on the finding that this sequence was sufficient to separate mTFP from the inner leaflet of the PM in the KIR system described above^24^,^25^. Indeed, the mIgG-Linker25-tail showed significantly decreased FRET efficiency compared with the WT mIgG-tail (Fig. 5b,c), confirming that the mIgG-Linker25-tail in the context of IgG-BCRs is in a solvent-exposed state in quiescent B cells.

### mIgG-Linker25-tail triggers inflated Ca^2+^ mobilization

We compared the activation strength of the IgG-BCR with a mIgG cytoplasmic tail that was either membrane-sequestered (mIgG-tail) or solvent-exposed (mIgG-Linker25-tail). For the following experiments, unless otherwise specified, we used stable Ramos B cell lines expressing similar levels of surface IgG-BCRs with either mIgG-tail or mIgG-Linker25-tail (Supplementary Fig. 2a). Additionally, we did not fuse a fluorescent protein to the C-terminus of the mIgG-tail or the mutant in these following functional studies to avoid affecting their ability to recruit Grb2 (ref. 16). Previous studies reported that the mIgG-tail is responsible for stronger Ca^2+^ mobilization in IgG-BCR expressing memory B cells compared to IgM-BCR expressing naive B cells^10^,^11^,^12^,^17^. Therefore, we compared Ca^2+^ mobilization in Ramos B cells stably expressing membrane-sequestered mIgG-tail or solvent-exposed mIgG-Linker25-tail. We also included a control (mIgG-tailless) with the cytoplasmic tail of mIgG swapped with the mIgM cytoplasmic tail for the purpose of mimicking the function of IgM-BCRs in naïve B cells (Fig. 5a). We measured calcium signals with the ratio-metric calcium probes Fluo-4-AM and Fura Red-AM. Strikingly, upon IgG-BCR crosslinking, the solvent-exposed mIgG-Linker25-tail exhibited dramatic hyper-activation with faster and stronger Ca^2+^ mobilization than the mIgG-tail (Fig. 5d). As a system control, the mIgG-tailless showed decreased Ca^2+^ mobilization response compared to the mIgG-tail, consistent with published results^10^,^11^,^12^,^17^. To exclude potential non-specific effects induced by the 25 aa linker sequence, we also tested mIgG-Linker25 which contained the 25 aa linker but not the cytoplasmic tail of mIgG (Fig. 5a). Not a surprise, the mIgG-Linker25 induced a much weaker Ca^2+^ mobilization response than the mIgG-tail (Supplementary Fig. 2b). This suggested that the inflated Ca^2+^ mobilization response of the mIgG-Linker25-tail was because of its solvent-exposed feature but not to the 25 aa linker. We also confirmed the inflated Ca^2+^ mobilization response of the mIgG-Linker25-tail compared with the mIgG-tail after stimulation with different concentrations of antigens (Fig. 5e). Strikingly, the hyper-reactive feature of the solvent-exposed mIgG-Linker25-tail was also evident at very-low antigen concentrations (Supplementary Fig. 2c). We conclude that the mIgG-tail can augment the Ca^2+^ mobilization response for IgG-BCR compared with IgM-BCR, and that the solvent-exposed mIgG-Linker25-tail is obviously more hyperactive than the membrane-sequestered mIgG-tail.

### mIgG-Linker25-tail induces excessive BCR signalling

**Figure 6:**
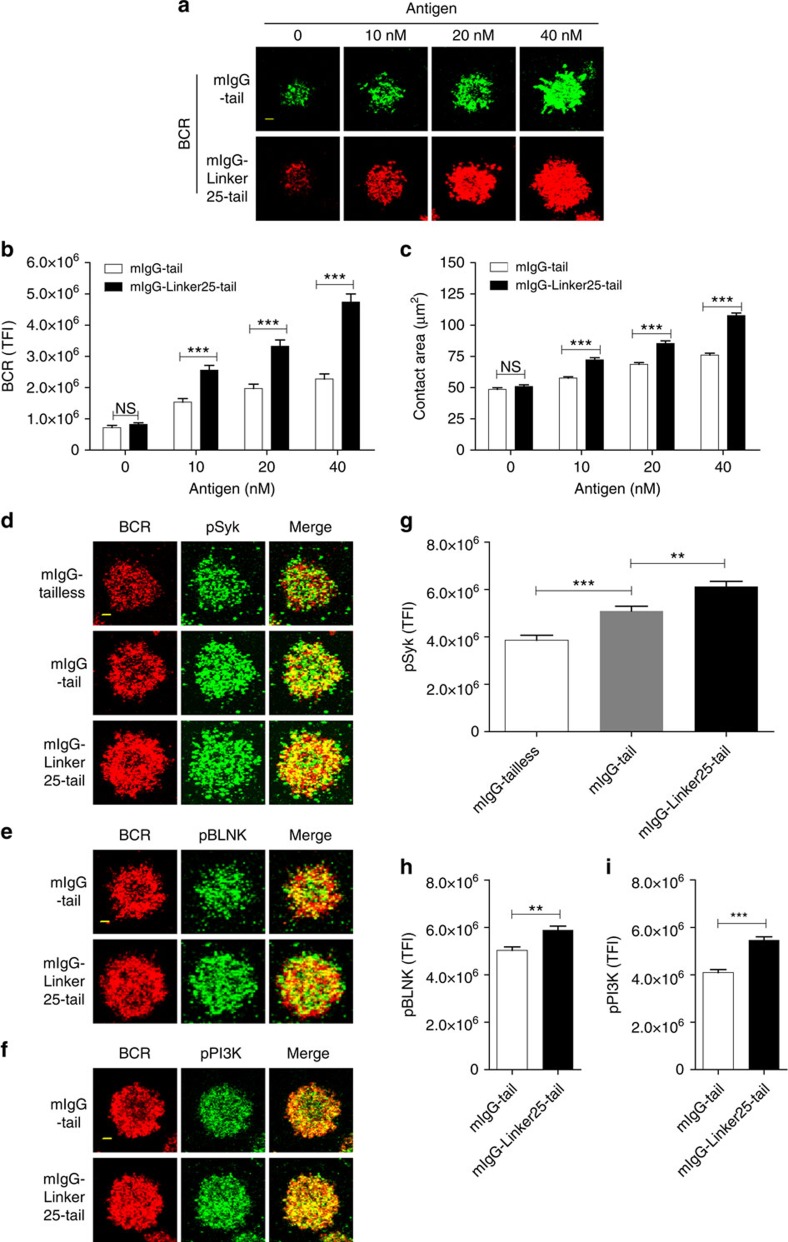
Solvent-exposed mIgG-tail induces the excessive recruitment of prominent BCR signalling microclusters into the immunological synapse. (**a**) TIRFM images to show the synaptic accumulation of BCRs in Ramos B cells expressing mIgG-tail or mIgG-Linker25-tail that were placed on surrogate antigens presenting PLBs. (**b**,**c**) Statistic quantification for the TFI of accumulated BCRs in the immunological synapse (**b**) and for the size of the contact area (**c**) of Ramos B cells expressing mIgG-tail and mIgG-Linker25-tail as represented in **a**. (**d**–**f**) Representative TIRFM images were given to show the synaptic recruitment of pSyk (**d**) pBLNK (**e**) pPI3K (**f**) in Ramos B cells expressing mIgG-tail or mIgG-Linker25-tail. (**g**–**i**). Statistic quantification were given to compare the synaptic accumulation of signaling molecules of pSyk (**g**) pBLNK (**h**) and pPI3K (**i**) in Ramos B cells expressing mIgG-tail or mIgG-Linker25-tail. In all the figures, given is one representative data out of three independent experiments. Bars indicate mean+s.e.m. Scale bar, 1.5 μm (**a**,**d**–**f**). Two-tailed *t*-tests were used for the statistical comparisons. ***P<0.001; **P<0.01.

All the above experiments were performed using soluble antigen to activate B cells. Membrane-bound antigens presented by antigen presenting cells are the predominant forms of antigens that B cells encounter *in vivo*^30^. Supported by the high-resolution total internal reflection fluorescence microscopy (TIRFM) imaging technique, a series of transient, dynamic and ordered membrane molecular events that occur during the initiation of membrane-bound antigen-induced B cell activation have been reported^8^,^15^,^31^,^32^,^33^. Thus, we compared these early events during the activation of Ramos B cells expressing the mIgG-Linker25-tail versus the mIgG-tail upon the recognition of membrane-bound biotinylated anti-mouse IgG surrogate antigens presented on a fluid planar lipid bilayer (PLB). Statistical quantification of the TIRFM images suggested that upon antigen recognition, the mIgG-Linker25-tail induced the recruitment of a significantly greater amount of BCR microclusters into the centre of the contact zone to form a larger B cell immunological synapse than the mIgG-tail (Fig. 6a–c). Moreover, the phosphorylation of downstream signalling molecules, including spleen tyrosine kinase (Syk), B cell linker (BLNK) and phosphatidylinositol-4,5-bisphosphate 3-kinase (PI3K), was significantly higher in the immunological synapse of B cells expressing mIgG-Linker25-tail than in that of B cells expressing mIgG-tail (Fig. 6d–i).

### mIgG-Linker25-tail induces hyper-proliferation of B cells

**Figure 7:**
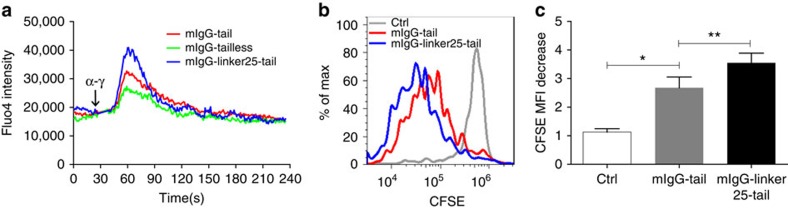
Solvent-exposed mIgG-tail induces the inflated Ca^2+^ mobilization and hyperproliferation in mouse primary B cells. (**a**) Analysis of the Ca^2+^ mobilization in mouse primary splenic B cells retrovirally expressing mIgG-tail, mIgG-tailless or mIgG-Linker25-tail. One representative data out of three independent experiments is shown. (**b**,**c**) A representative proliferation profile was given in mouse primary B cells retrovirally expressing mIgG-tail or mIgG-Linker25-tail indicated by the intensity decrease of proliferation marker CFSE. In (**c**) given is the statistic comparison of the proliferation of primary B cells expressing mIgG-tail or mIgG-Linker25-tail by calculating the mean fluorescence intensity decrease fold of CFSE. Bars indicate mean+s.d. from three independent experiments. Two-tailed *t*-tests were used for the statistical comparisons. **P<0.01; *P<0.05.

All the above experiments were performed with B cell lines. Next, we attempted to confirm the key conclusions in mouse primary B cells. We first infected primary splenic B cells isolated from C57BL/6 mice with a standard pMSCV-based retroviral expression system. This system has a dual-promoter which drives the expression of the fluorescent protein mAmetrine along with the γ immunoglobulin heavy chain in the format of either mIgG-tail or mIgG-Linker25-tail (Supplementary Fig. 4). Upon stimulation with anti-mouse IgG (α-γ), an inflated Ca^2+^ mobilization response was consistently observed in the mIgG-Linker25-tail expressing primary B cells compared with the mIgG-tail expressing primary B cells (Fig. 7a). In addition to inflated Ca^2+^ mobilization, enhanced proliferation is the other key feature of IgG-BCR expressing memory B cells during antigen recall. We therefore compared proliferation of mIgG-tail or mIgG-Linker25-tail expressing mouse primary B cells after antigen stimulation. Cell proliferation was monitored by detecting the fluorescence dilution of the proliferation marker carboxy fluoresceindiacetate succinimidyl ester (CFSE). Strikingly, the mIgG-Linker25-tail expressing primary B cells exhibited significantly faster proliferation than the mIgG-tail expressing primary B cells (Fig. 7b,c). This indicates that the solvent-exposed mIgG-Linker25-tail augments the proliferation rate of mouse primary B cells compared with the membrane-sequestered mIgG-tail.

### Physiological clues dissociate mIgG-tail from the PM

**Figure 8:**
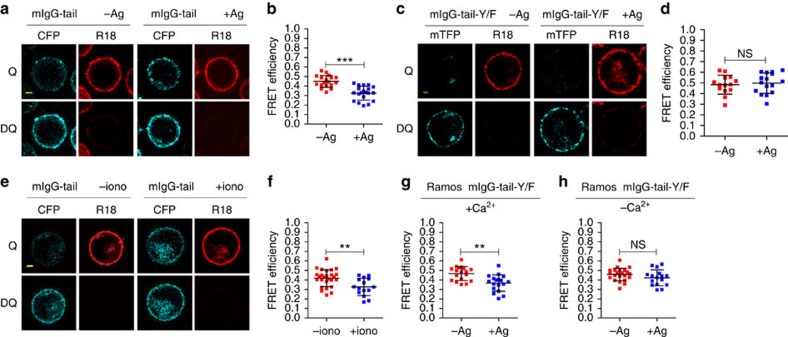
Antigen engagement or Ca^2+^ mobilization independently facilitates the dissociation of mIgG-tail from the PM. (**a**–**d**) Dequenching FRET performed after crosslinking of extracellular domain of mIgG by NP8-BSA in J558L cells expressing B1-8-IgG-BCRs with mIgG-tail (**a**,**b**) or mIgG-tail-Y/F (**c**,**d**). (**e**,**f**) FRET analysis to evaluate the function of Ca^2+^ influx in dissociating mIgG-tail from the PM in J558L cells expressing B1-8-IgG-BCRs. (**g**,**h**) Dequenching FRET analysis of Ramos cells expressing IgG-BCR with mIgG-tail-Y/F in sample buffer with Ca^2+^ (**g**) or without Ca^2+^ (**h**). For all the analysis, Scale bar, 1.6 μm. Each dot represents one cell analysed in three independent experiments. Bars indicate mean±s.d. Two-tailed *t*-tests were used for the statistical comparisons. ***P<0.001; **P<0.01; NS, not significant.

We investigated the dynamic interaction between the mIgG-tail and the PM in activated B cells in response to antigen engagement. For this we used FRET analysis with CFP or mTFP as the FRET donor, and R18 as the FRET acceptor. We first looked at the effect of antigen engagement on the membrane binding of mIgG-tail. Ca^2+^ has been reported to be able to disrupt the membrane binding of TCR cytoplasmic tails^25^. To distinctly separate the effect of Ca^2+^ from the antigen engagement of IgG-BCR, we used J558L cells that are deficient of BCR-induced Ca^2+^ mobilization^34^,^35^, to express NP-specific B1-8-IgG-BCRs. Moreover, we used Ca^2+^-free buffer in the FRET experiments to exclude the contribution of externally influxed Ca^2+^ if any in the FRET assay. When stimulated with soluble NP8-BSA antigens, B1-8-IgG-BCR expressing J558L cells showed evident BCR patching on the PM (Fig. 8a,c), indicating that the B1-8-IgG-BCRs was efficiently crosslinked by NP8-BSA. Importantly, the FRET efficiency between mIgG-tail and the PM in activated cells decreased significantly compared with the unstimulated control (Fig. 8b). This observation supported the notion that antigen engagement can be the initial trigger for the dissociation of the membrane-sequestered mIgG-tail from the PM and such trigger is obviously independent on Ca^2+^ mobilization. The phosphorylation of ITT-tyrosine occurs upon antigen engagement^17^. The phosphate group on ITT-Tyrosine will introduce negative charges to mIgG-tail, which most likely prevents the re-binding of ITT to acidic phospholipids due to charge repulsion. To assess the importance of the phosphorylation of ITT in the dissociation event of mIgG-tail from the PM upon antigen engagement, we constructed ITT-Tyrosine mutant mIgG-tail-Y/F and performed the FRET experiments similarly as above in Ca^2+^-free buffer. We observed that mIgG-tail-Y/F failed to dissociate from the PM after antigen engagement (Fig. 8c,d). These results suggest that the phosphorylation of ITT upon antigen engagements is required for stabilizing mIgG-tail in the cytosol when Ca^2+^ mobilization is not available.

Ca^2+^ mobilization is one of the most important early events in BCR signalling^15^,^25^,^36^. In the literature, externally influxed Ca^2+^ from the calcium release-activated channel (CRAC) is shown to neutralize the negatively charged acidic phospholipids within the inner leaflet of the PM^25^. Therefore we studied this lipid-modification effect of Ca^2+^ on the interaction between mIgG-tail and the PM in activated B cells. J558L cells that express B1-8-IgG-BCRs were stimulated with ionomycin, a potent and selective Ca^2+^ ionophore, to induce Ca^2+^ influx in cells without crosslinking the IgG-BCRs. We found that the FRET efficiency between the mIgG-tail and PM decreased significantly in cells treated with ionomycin compared with the non-treated control cells (Fig. 8e,f), demonstrating that exogenously influxed Ca^2+^ can independently lead to the dissociation of mIgG-tail from the inner leaflet of the PM even in the absence of IgG-BCR engagement. This conclusion was also supported by *in vitro* FP assay showing that Ca^2+^ can disrupt the binding between mIgG-tail and the acidic lipid bicelles (Supplementary Fig. 5).

Having clarified the independent contribution of either antigen engagement or Ca^2+^ mobilization in driving the dissociation of mIgG-tail from the PM, we next addressed the potential sequential order of these two driving forces in dissociating mIgG-tail from the PM at the physiological condition of inducing Ca^2+^ mobilization by antigen engagement rather than at the non-physiological condition by ionomycin. To address this question, we expressed the abovementioned mIgG-tail-Y/F construct in human Ramos B cells. Different from J558L cells, Ramos B cells are competent to induce strong Ca^2+^ mobilization upon BCR crosslinking^17^. We similarly performed FRET experiments in either Ca^2+^-containing buffer (2 mM Ca^2+^) or Ca^2+^-free buffer. The results indicate that antigen engagement can induce the dissociation of mIgG-tail-Y/F from the PM in Ca^2+^-containing buffer (Fig. 8g), suggesting that the phosphorylation of ITT-tyrosine is dispensable for the dissociation of mIgG-tail from the PM when influxed Ca^2+^ is available. In the experimental condition of using Ca^2+^-free buffer there was a lack of sufficient dissociation of mIgG-tail-Y/F from the PM as shown by no significant FRET efficiency changes upon antigen engagement (Fig. 8h), which further supports the conclusion that ITT phosphorylation is required for maintaining the solvent-exposed state of mIgG-tail in the initial BCR signalling stage before the Ca^2+^ mobilization.

**Figure 9:**
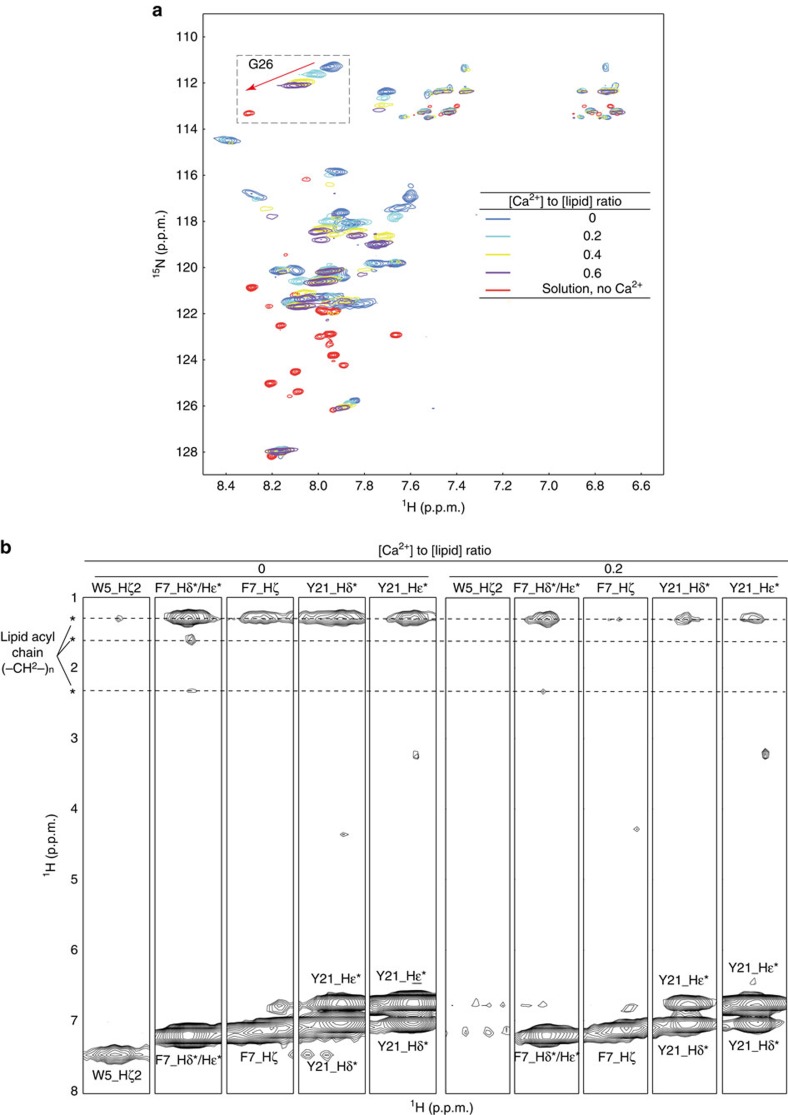
Ca^2+^ induced the solvent exposure of the signalling tyrosine in the ITT motif. (**a**) Superimposed ^15^N–^1^H TROSY spectra of mIgG-tail with POPG bicelles, mIgG-tail with POPG bicelles and different concentrations of Ca^2+^, and mIgG-tail in solution as a control. Membrane-sequestered mIgG-tail and solvent-exposed mIgG-tail had very different amide resonance spectra. Ca^2+^ was titrated into the mIgG-tail with POPG sample at a molar ratio of [Ca^2+^]: [POPG] from 0 to 0.5. In response to the increase in the Ca^2+^ concentration, the mIgG-tail amide resonances exhibited systematic shifts from the membrane-bound state to the solvent-exposed state. G26 is shown as an example of Ca^2+^ titration effects (upper dotted box). (**b**) Strips from aromatic NOESY spectra showing NOEs (distance of <5 Å) between the aromatic protons of W5, F7 and Y21 and the methylene protons in the lipid acyl chains. The substantial intermolecular NOE signals (marked by asterisks) observed in the absence of Ca^2+^ indicated the insertion of tryptophan, phenylalanine and tyrosine side chains into the membrane hydrophobic interior. The addition of Ca^2+^ resulted in the significant decrease of NOE signals, which indicated that side chains of these three residues were dissociated from the membrane core region.

To further study the effect of Ca^2+^ at atomic resolution, we used NMR to examine how Ca^2+^ regulates the interaction between the mIgG-tail and the PM. We titrated a ^15^N-labelled mIgG-tail in the presence of acidic lipid POPG bicelles with increasing concentrations of Ca^2+^, and measured the 2D ^15^N–^1^H TROSY spectra. Upon the increase of Ca^2+^ concentrations, there were substantial changes in both chemical shifts and intensities in the whole spectra. The mIgG-tail showed obvious tendency toward the solvent-exposed state (Fig. 9a). We further performed NOE analysis of the ^13^C, ^15^N-labelled mIgG-tail with POPG bicelles in the presence of Ca^2+^. Importantly, we found that the addition of Ca^2+^ led to the significant reduction of Y21-lipid NOE signals (Fig. 9b), confirming that Ca^2+^ induces the solvent exposure of the signalling tyrosine in the ITT motif. In conclusion, all these results demonstrate that antigen engagement or Ca^2+^ mobilization can independently induce the dissociation of the mIgG-tail from the PM.

## Discussion

Memory B cells that express isotype-switched IgG-BCRs usually undergo rapid activation and swift proliferation to account for the rapid and high-titred memory IgG antibody response upon antigen recall. Numerous studies have investigated the mechanism of the enhanced activation of the IgG-BCR: (1) placing an evolutionarily conserved sequence harbouring the unique ITT motif in the mIgG-tail that recruits Grb2 upon phosphorylation (pITT)^17^, which in turn could enhance Btk recruitment and lower the activation threshold of PLCγ2^16^; (2) changing the equilibrium of the activating versus inhibitory function of Grb2 mediated by its interaction with Btk versus Dok-3^16^; (3) recruiting synapse associated protein 97 (SAP97) to enhance the microclustering of IgG-BCRs^7^; (4) reducing the expression of the key transcription factor Bach2 to accelerate the proliferation and differentiation to plasma cells^37^. Thus, it has been proposed that the IgG-BCR can be characterized as an ‘effector' receptor that can undergo quick initiation of activation. In contrast, the IgM-BCR can be characterized as an ‘affector' receptor that needs to go through multiple checkpoints before the activation decision is made^38^. However, it is not clear how IgG-BCR appropriately ensures the signalling hierarchy of utilizing ITT signalling to amplify ITAM signalling in response to antigen stimulation.

Here, we provide multiple lines of evidences to demonstrate that acidic phospholipids in the PM play a crucial role in regulating IgG-BCR signalling hierarchy. Ionic interactions between the positively charged mIgG-tail and the negatively charged acidic phospholipids can efficiently quench the signalling capability of the key ITT-Tyrosine of mIgG-tail in quiescent B cells by sequestering the ITT-Tyrosine within the membrane bilayer. These results mirror the observations from TCR studies, which showed that the ITAM tyrosines on the cytoplasmic tail of CD3ɛ are protected by the hydrophobic core of the PM^24^. Mechanistically, we show that basic residues in mIgG-tail are critical for its association with the PM. It is worth noting that the cytoplasmic domains of Igα and Igβ, which are negatively charged, may account for their lack of interactions with the inner leaflets of the PM as recently reported^39^.

We designed a mIgG-Linker25-tail mutant to evaluate the function of IgG-BCR with a solvent-exposed mIgG-tail in comparison to the WT IgG-BCR with a membrane-sequestered mIgG-tail. The most striking finding from the experiments using mIgG-Linker25-tail is that such a solvent-exposed mIgG-Linker25-tail in the context of the IgG-BCR leads to a more aggressive Ca^2+^ mobilization response and excessive recruitments of prominent BCR signalling microclusters into the B cell immunological synapse in response to antigen stimulation, which eventually inflates the hyperproliferation of B cells compared to WT mIgG-tail. These data suggest that membrane-sequestered conformation of the cytoplasmic ITT motif grants a mechanism for the ordered signalling hierarchy for the activation of IgG-BCR expressing memory B cells. Our model predicts that in quiescent memory B cells, the ITT-Tyrosine in mIgG-tail is protected within the lipid microenvironment of the PM to avoid the disordered signalling hierarchy.

We demonstrated that antigen engagement and Ca^2+^ mobilization as two driving forces can independently dissociate the mIgG-tail from the PM. Antigen engagement of IgG-BCRs will likely trigger the transient dissociation of mIgG-tail from the PM. In previous studies of TCR, receptor clustering and local lipid environment change are shown to contribute to the dissociation of ITAMs from the PM^40^,^41^. These mechanisms might also apply to IgG-BCR. Notably, we found that ITT phosphorylation is required to stabilize mIgG-tail at solvent-exposed state in the initial BCR signalling stage when Ca^2+^ mobilization has not been triggered. We also found that Ca^2+^ influx alone is sufficient to trigger the dissociation of mIgG-tail from the PM in an antigen-independent manner.

**Figure 10:**
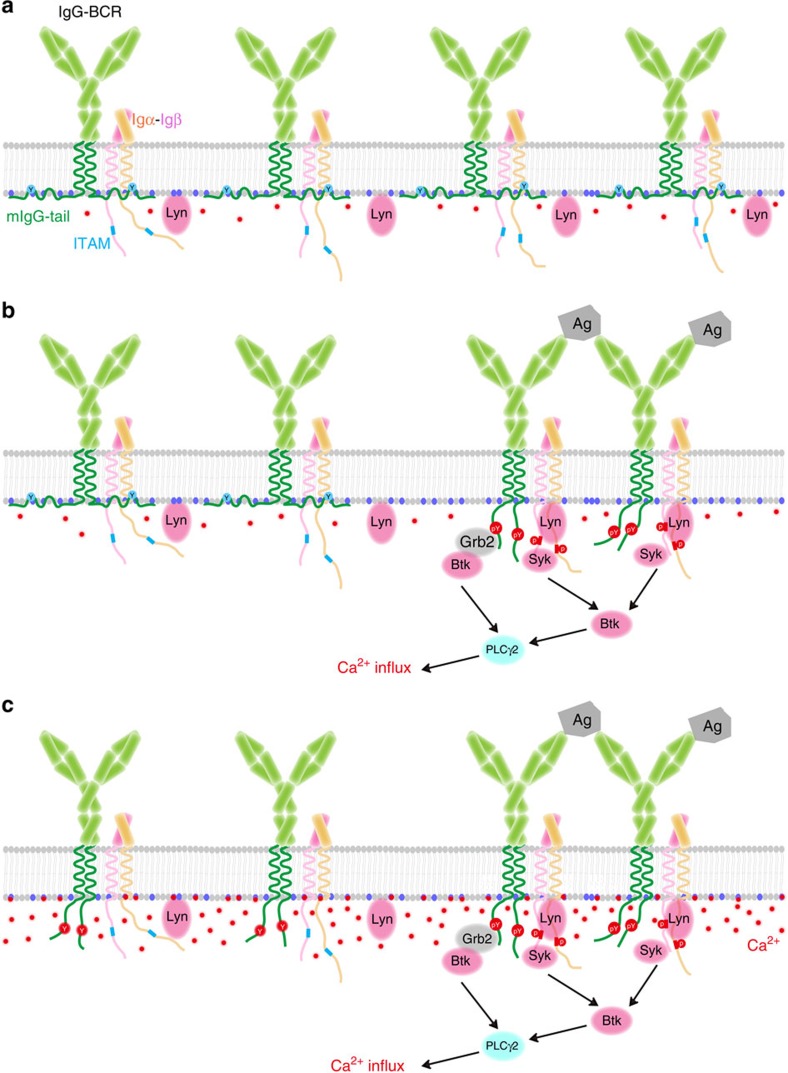
Dynamic interaction between mIgG-tail and acidic phospholipids governs the enhanced activation of memory B cells. (**a**) In quiescent IgG-BCR expressing memory B cells, the positively charged mIgG-tail interacts with acidic phospholipids in the inner leaflet of the plasma membrane, leading to the sequestration of the key ITT tyrosine within the membrane hydrophobic core. (**b**) Upon the antigen engagement, Igα- and Igβ-ITAMs are phosphorylated by Lyn, and then recruit downstream signalling molecules to induce Ca^2+^ mobilization. Meanwhile, the antigen engagement of IgG-BCRs also initially perturbs the interaction of the ITT with the PM. Such disturbance triggers the exposure of the ITT-tyrosine and the subsequent phosphorylation by Syk. Phosphorylated ITT carries extra negative charges. The charge repulsion from acidic lipids will make the pITT stably localized in cytosol to recruit Grb2, thus further enhancing the Ca^2+^ influx^16^,^17^. (**c**) ITAM signalling triggers Ca^2+^ influx through CRAC channel. The accumulation of membrane-proximal Ca^2+^ can neutralize the negative charges of acidic phospholipids and thus can in turn facilitate the dissociation of mIgG-tail from the plasma membrane. Thus, all these steps in (**a**–**c**) work in an ordered manner to amplify the antigen-initiated signalling to a greater magnitude and thus contribute to the enhanced activation of IgG-BCR expressing memory B cells in comparison with the case of IgM-BCR expressing mature naïve B cells.

Based on all these data, we propose that there shall be multi-layers of forces accounting for the efficient exposure of the ITT-Tyrosine in mIgG-tail from the PM at physiological condition. These forces include: (1) direct antigen engagement; (2) phosphorylation of the ITT-Tyrosine; (3) the exogenously influxed Ca^2+^ through the CRAC. Our model (Fig. 10) is that in quiescent IgG-BCR expressing memory B cells, mIgG-tail interacts with the inner leaflet of the PM to sequester key ITT motif within the membrane hydrophobic core. Antigen engagement will initially perturb the interaction of the ITT with the PM in a certain extent. Such disturbance would likely transiently trigger the exposure of the ITT from the membrane-sequestered state. Under conditions where the transiently exposed ITT-Tyrosine can be phosphorylated by Syk, the negative charges from the phosphorylated tyrosine will efficiently stabilize the phosphorylated ITT in a solvent-exposed state. Concurrently, antigen engagement swiftly induces Ca^2+^ influx through the conventional signalling module ITAMs on the cytoplasmic domain of Igα and Igβ. The exogenously influxed Ca^2+^ through the CRAC can efficiently expose mIgG-tail of IgG-BCRs. Since it is known that the ITT signalling can further enhance Ca^2+^ mobilization, which in turn can activate more IgG-BCRs^16^,^17^, this type of positive feedback regulation can potently amplify the initial antigen-stimulated BCR signalling to a higher amplitude. In our model (Fig. 10), we also speculate that the bystander IgG-BCRs that are not necessarily engaged with antigens might also have the chances to be dissociated from the PM by exogenously influxed Ca^2+^ through the CRAC. Such a speculation is supported by the reports showing the antigen-independent activation of bystander IgG-BCRs in both mouse and human IgG-BCR expressing primary B cells, in which IgG-BCR microclusters grow faster compared to antigen microclusters^15^,^42^. In contrast, upon antigen stimulation, the IgM-BCR microclusters grow at a comparable level to the antigen microclusters, suggesting a lack of antigen-independent activation for IgM-BCRs^15^,^42^.

In conclusion, the findings presented here indicate a mechanism for the ordered signalling hierarchy in the initiation of the activation of IgG-BCR expressing memory B cells. Such a mechanism is achieved by membrane sequestration of the key ITT motif of the mIgG-tail in quiescent B cells and the dynamic exposure of the ITT motif upon antigen engagement and Ca^2+^ mobilization.

## Methods

### Peptides and reagents

CP488-mIgG-tail (C-(CP488 dye conjugated)-GGAKVKWIFSSVVELKQTLVPEYKNMIGQAP), CP488-mIgG-tail-K/S (C-(CP488 dye conjugated)-GGA**S**V**S**WIFSSVVEL**S**QTLVPEY**S**NMIGQAP) and mIgG-tail (KVKWIFSSVVELKQTLVPEYKNMIGQAP) peptides were purchased from California Peptides (Napa, CA) and CPC scientific (Sunnyvale, CA). All these peptides were purified by high-performance liquid chromatography and verified purity of >90% was obtained by mass spectrometry. Biotinylated F(ab)_2_ fragment goat anti-mouse IgG (115-066-071), and goat anti-mouse IgG, Fc specific (115-006-071) were from Jackson ImmunoResearch. Fab anti-mouse IgG, Fc specific (Fab anti-mouse IgG) was acquired by processing whole IgG of anti-mouse IgG with a Fab micro preparation kit (Pierce). Fab anti-mouse IgG antibodies were conjugated to Alexa fluorophores using Alexa Fluor mAb labelling kits (Invitrogen). Lipids (POPG, POPS, POPC and DHPC) were purchased from Avanti polar lipids.

### Cells and plasmids

Human Ramos B cell line and mouse B cell lines J558L, A20, CH27 were gifts from Dr Susan K. Pierce (NIAID, NIH, USA) and maintained in complete RPMI-1640 medium with 10% heat-inactivated fetal bovine serum and antibiotics. J558L cells stably expressing NP-specific B1-8-mIgM-CFP or mIgG-CFP were constructed by electrotransfection and antibiotics selection^8^. The expression plasmids for KIR-3aa-mTFP, KIR-25aa-mTFP, KIR-mIgG-tail-mTFP and IgG-BCR-mIgG-tail were in pHAGE backbone. All the mIgG-tail variant vectors were constructed based on their WT version. Stable A20 or Ramos cells expressing WT or mutated mIgG-tail were acquired by three vectors (pHAGE, psPAX2 and pMD2.G) based lentivirus transduction and flow cytometry sorting. The expression vectors of IgG-BCR with mIgG-tail, mIgG-tailless or mIgG-Linker25-tail for primary B cells transduction were in pMSCV backbone with dual-promoter driven fluorescent protein mAmetrine.

### Bicelle preparation

Bicelles (*q*=0.8) were prepared by mixing the long-chain phospholipids POPG (1-Palmitoyl-2-oleoyl-phosphatidylglycerol) or POPC (1-palmitoyl-2-oleoyl- phosphatidylcholine) with the short-chain phospholipid DHPC (dihexanoyl- phosphatidylcholine) at a ratio of 0.8:1 (POPG/POPC:DHPC) in 20 mM Bis-Tris buffer (pH 6.7). The mixture was subjected to several freeze-thaw cycles with vortexing until the lipids were fully dissolved^24^,^25^.

### Fluorescence polarization (FP) assay

To detect the binding kinetics of mIgG-tail WT or Mut peptide to phospholipids, 100 nM CP488 dye conjugated peptides and different concentrations of POPG, POPS, POPC and mixture lipids bicelles were incubated in a 384-well plate for 15 min at room temperature (RT) away from light. To measure the inhibitory function of Ca^2+^ for the binding, 100 nM CP488 dye conjugated peptides and 2.5 mM POPG bicelles were incubated as above described and different concentrations (0–10 mM) of Ca^2+^ were added to the peptide-phospholipid interaction system. FP values were measured by a Tecan Infinite M1000Pro Microplate Reader. Sample buffer (20 mM HEPES, pH 7.0, 150 mM NaCl) was used in the FP experiments to detect mIgG-tail WT peptide binding to different lipid bicelles. Sample buffer (50 mM Tris-HCl, pH 7.4, 150 mM NaCl) was used in the FP experiments to compare the binding of either mIgG-tail WT or K/S Mutant to lipid bicelles, and such sample buffer was also used in the FP experiments to detect the inhibitory function of Ca^2+^ for the binding.

### Tryptophan fluorescence emission spectrum (TFES) assay

To measure the TFES of mIgG-tail WT and Mut, 10 μM peptides and 0–2 mM POPS, POPC or mixture lipids bicelles were mixed and incubated for 20 min at RT and a Varian Cary Eclipse machine was used with the excitation wavelength 290 nm and emission wavelength 300–400 nm. The sample buffer was 50 mM Tris-HCl, pH 7.4, 150 mM NaCl.

### FRET measurement

To perform the FRET experiments, 1 × 10^6^ cells were collected and washed once with 1 × PBS, and then resuspended in 1 ml cell buffers. Before loading to the poly-L-lysine (Sigma) coated chambered coverglass slides (Thermo Fisher Scientific), 250 μl aliquot of cells were stained with 300 nM octadecyl rhodamine B (R18) (Invitrogen) on ice for 3 min. The chamber was then mounted onto a Nikon A1RSi microscope and maintained for 5 min to allow cell adhesion. Dequenching FRET images were captured^24^,^25^. mTFP or CFP was excited with Argon 457 nm laser line and visualized using the 515/30 bandpass filter. R18 was excited with the Solid State 561 nm laser line, visualized using the 595/50 bandpass filter. For the antigen stimulation FRET experiments, the cell buffer is Mg^2+^ and Ca^2+^ free Ringer's buffer (155 mM NaCl, 4.5 mM KCl, 10 mM D-glucose, 10 mM HEPES, pH 7.4) and cells were stimulated for 5 min with 10 μg ml^−1^ NP8-BSA at RT before imaging. For the ionomycin stimulation FRET experiments, the cell buffer is Mg^2+^ free Ringer's buffer with 2 mM Ca^2+^ and images were taken immediately after adding 5 μM ionomycin (Sigma) to the chamber. For the antigen stimulation FRET experiments in mIgG-tail-Y/F expressing Ramos or J558L cells, the cell buffer is Ringer's buffer with or without 2 mM Ca^2+^ and cells were stimulate for 5 min with 15 μg ml^−1^ F(ab)_2_ anti-mouse IgG (for Ramos cells) or 10 μg ml^−1^ NP8-BSA (for J558L cells) at RT before imaging. All the other FRET experiments were performed in Ringer's buffer with 2 mM Ca^2+^ and 1 mM Mg^2+^. All the images were processed with Image J (NIH, USA). FRET efficiency was calculated with the formula FRET efficiency=(DQ-Q)/DQ, DQ and Q represented dequenched and quenched donor fluorescence intensity respectively.

### Circular dichroism

The secondary structures of the mIgG-tail in solution or upon lipid binding were examined by the far UV CD. Spectra were recorded from 190–250 nm at 16 °C in a 0.1 cm path length quartz cuvette using a J-715 spectrometer (JASCO Corporation). The experimental samples contained 20 μM mIgG-tail with or without 0.5 mM POPG or POPC bicelles in 20 mM Tri-HCl buffer (pH 7.4). Control sample without mIgG-tail but containing all other components were recorded before each experimental sample to set the baseline.

### Expression and purification of mIgG-tail peptide

The murine mIgG-tail was expressed as a His_6_-SUMO fusion protein using pET-28b vector (EMD Biosciences). Transformed *Escherichia coli* strain BL21 (DE3) cells were cultured in M9 minimal medium supplemented with one or more stable isotope labels to generate labelled proteins for NMR studies. Protein expression was induced at OD600 ∼0.6 with 0.1 mM IPTG for overnight (minimal medium) at 37 °C. Fusion protein was first purified by a nickel-chelating affinity chromatography (Roche), followed by SUMO protease Ulp1 treatment and dialysis overnight in a Tris-HCl buffer, pH 8.0 at 4 °C to cleave the His_6_-SUMO tag and to remove the imidazole. The enzyme was added at a ratio of 1:200 (w/w) of the fusion protein. The His_6_-SUMO tag was later removed via a second nickel-chelating chromatography. Protein was further purified by reverse-phase high-performance liquid chromatography in two steps using both semi-preparative and analytical C18 columns (Agilent). The identity of purified mIgG-tail was confirmed by MALDI-TOF mass spectrometry.

### NMR Spectroscopy

All NMR experiments were performed on Bruker AVANCE 600MHz and Agilent ASC 800 MHz spectrometers equipped with cryogenic probes at 35 °C. TROSY experiments were performed with 0.1 mM ^15^N-labelled mIgG-tail and 20 mM POPG or POPC bicelles (*q*=0.8, *q* is the molar ratio of long-chain lipids (POPG or POPC) to short-chain lipids (DHPC)) in 20 mM Bis-Tris buffer (pH 6.7). All spectra were processed using the program NMRPipe^42^ and analysed by NMRView^43^ and KUJIRA^44^.

### Aromatic NOESY experiments

Because of the low concentration of the protein/lipid/Ca^2+^ mixture samples, 2D proton NOESY experiments were performed to detect NOEs from mIgG-tail aromatic protons to lipid methylene protons. For the indirect dimension, background signals from mIgG-tail were suppressed by double ^13^C filtering, and only lipid methylene protons evolved. For the direct dimension, the aromatic protons were selected by aromatic ^13^C edition. The samples were diluted in 90% D_2_O to achieve better water suppression and reduce the spin diffusion effect. NOESY experiments were performed with a NOE mixing time of 120 ms, 120 points in the indirect dimension, 2,048 points in the direct dimension and 1,024 accumulated transients. NOESY experiments were performed with 0.1 mM ^13^C, ^15^N-labelled mIgG-tail, 20 mM POPG bicelles (*q*=0.8) and 0 or 4 mM Ca^2+^ in 20 mM Bis-Tris buffer (pH 6.7).

### Ca^2+^ mobilization analysis

To detect the intracellular Ca^2+^ mobilization of B cells, cells were processed with 2 μg ml^−1^ Fluo4-AM (Invitrogen) and 3 μg ml^−1^ Fura Red-AM (Invitrogen) at 30 °C for 20 min with 2.5 mM probenecid (Invitrogen) in HBSS buffer (with 1.26 mM Ca^2+^) with 1% FBS. Then cells were washed with HBSS once and incubate at 37 °C for another 20 min in HBSS buffer(with 1.26 mM Ca^2+^) with 1% FBS. After basal level of Ca^2+^ concentration was monitored for 30 s, cells were stimulated with different antigens. The fluorescence of Fluo-4 and Fura Red were measured on BD Accuri C6 or LSR II cytometer and ratio of Fluo-4 to Fura Red was calculated in FlowJo (Tree Star).

### Retroviral infection of mouse primary splenic B cells

Mouse primary splenocytes were isolated from C57BL/6 mice, and after 24 h incubation with 10 μg ml^−1^ lipopolysaccharide (LPS, Sigma) in RPMI-1640 medium containing 10% FBS, penicillin, streptomycin and 50 μM β-mercaptoethanol, 4 × 10^6^ splenic cells were spin-infected for 3.5 h with 1.5 ml concentrated retrovirus supernatants from Plat E cells transfected with pMSCV expression vector encoding the γ immunoglobulin heavy chain with mIgG-tail or mIgG-Linker25-tail. At 24 h after retrovirus infection, positive rate was detected by flow cytometry and the infection efficiency usually reached 30–50%. Positive primary B cells were used for the Ca^2+^ mobilization and proliferation analysis.

### Proliferation analysis of mouse primary splenic B cells

Positive primary B cells after retroviral infection were stained with 1 μM CFSE (Invitrogen) at 37 °C for 10 min and then seeded in 24-well plates with 500 μl medium containing 10 μg ml^−1^ F(ab)_2_ fragments anti-mouse IgG, Fc specific, 50 μM β-mercaptoethanol and 20 ng ml^−1^ rm-IL-4. After stimulation for 96 h, CFSE fluorescence intensity was detected by flow cytometry and data was analysed by FlowJo (Tree Star). CFSE mean fluorescence intensity of mAmetrine positive cells was normalized to that of mAmetrine negative cells from the same wells.

### Molecule imaging by TIRFM

PLBs containing biotinylated anti-mouse Ig surrogate antigen were prepared following our published protocols^7^,^8^,^15^,^32^. B cells were firstly stained with Alexa Fluor 647 conjugated Fab anti-mouse IgG, Fc specific and then loaded to the chamber to react with surrogate antigens on the PLBs for 10 min followed by 4% paraformaldehyde (PFA) fixation. TIRFM images were captured by an Olympus IX-81 microscope supported by Andor iXon+ DU-897D electron-multiplying CCD camera, Olympus 100 × 1.49 NA objective lens and a TIRF port. TIRFM image acquisition was controlled by Metamorph software (Molecular Devices) and the exposure time for 512 × 512 pixels image was 100 ms. Total fluorescence intensity (TFI) of BCRs and BCR downstream signalling molecules accumulated to the IS were statistically analysed based on the intensity and area by Image J (NIH, USA)^15^,^32^.

### Intracellular immunofluorescence staining experiments

Intracellular immunofluorescence staining of the signalling molecule in the IS of activated B cells were performed according to our published protocols^7^,^15^,^32^. Briefly, B cells were fixed with 4% PFA fixation for 30 min at RT. After washed with 1 × PBS, B cells were permeabilized with 0.2% Triton X-100 for 20 min and then blocked with 100 μg ml^−1^ goat non-specific IgG (Jackson ImmunoResearch Laboratory, 005-000-003) for 1 h at RT. Subsequently, cells were incubated with various primary antibodies including Anti-phospho-Syk (pY525/526) Ab (Cell Signaling, #2711, 1:500 dilution), Anti-phospho-PI3K p85 (pY458)/p55 (pY199) Ab (Cell Signaling, #4228, 1:500 dilution) and Anti-phospho-BLNK Ab (Biodragon, 1:500 dilution) at 37 °C for 1 h and then Alexa Fluor 568 conjugated F(ab)_2_ fragment goat anti- rabbit IgG (Invitrogen, 1:2,000 dilution) was used as the secondary antibody. TIRFM Images were processed with Image J (NIH, USA)^7^,^8^,^15^.

## Additional information

**How to cite this article:** Chen, X. *et al*. Acidic phospholipids govern the enhanced activation of IgG-B cell receptor. *Nat. Commun*. 6:8552 doi: 10.1038/ncomms9552 (2015).

## Supplementary Material

Supplementary Information Supplementary Figures 1-5
