# Supplementary material for: Acidic phospholipids govern the enhanced activation of IgG-B cell receptor
Source: Nat Commun. 2015 Oct 6;6:8552. doi: 10.1038/ncomms9552 (PMC4600742; doi:10.1038/ncomms9552)
Supplement: Supplementary Information — Supplementary Figures 1-5 [file ncomms9552-s1.pdf]

Supplementary Figures

Supplementary Fig. 1:

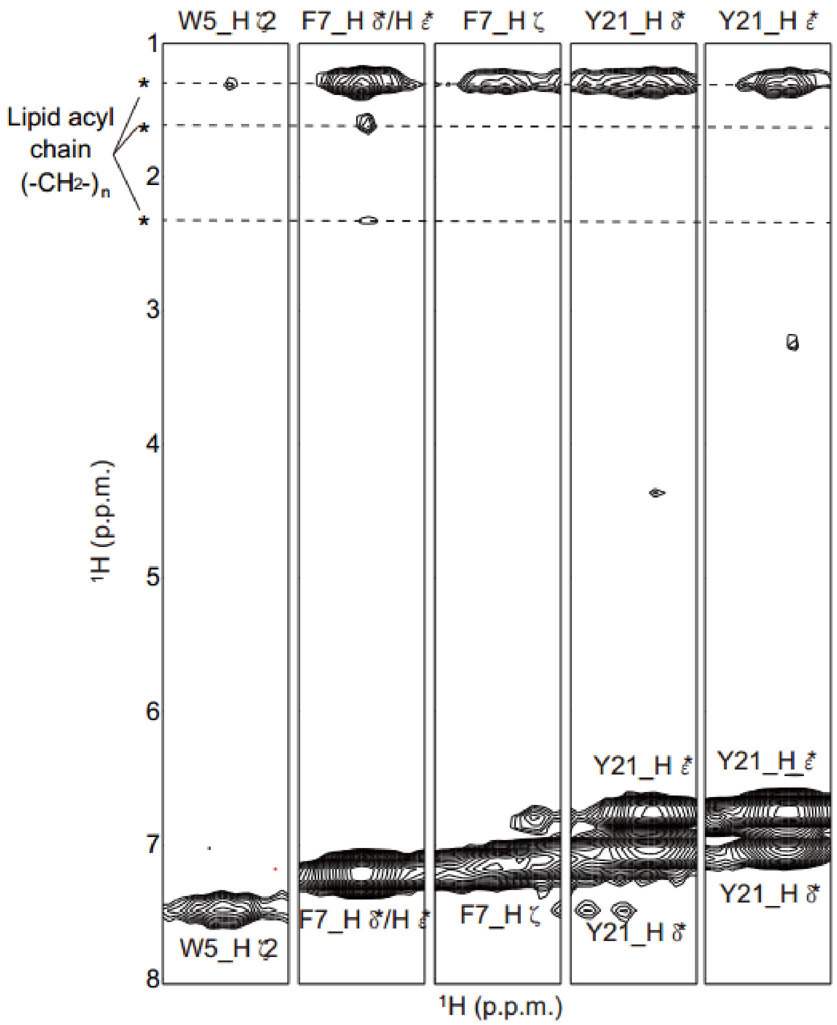

Supplementary Fig. 1: Lipid binding sequesters the signaling tyrosine of the mIgG-Tail within the hydrophobic core of the PM

Aromatic NOESY spectra showing NOEs between the aromatic protons of W5, F7 and Y21 in the mIgG-tail and the methylene protons of the lipid acyl chains in POPG bicelles. The substantial intermolecular NOE signals (marked by asterisks) observed indicated the insertion of tryptophan, phenylalanine and tyrosine side chains into the membrane hydrophobic interior.

Supplementary Fig. 2:

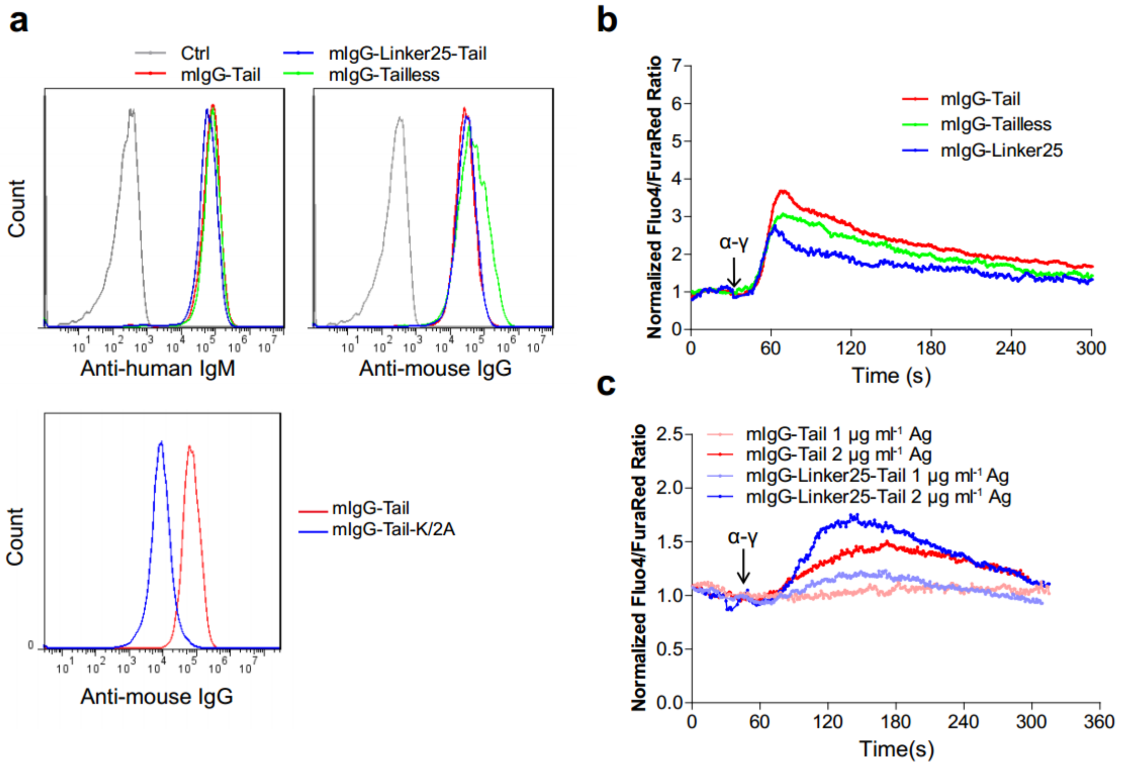

Supplementary Fig. 2: Solvent-exposed mIgG-Tail leads to inflated Ca^{2+} mobilization

(a). Given was the quantification of surface expressed IgG-BCRs in laboratory B cells stably expressing reconstructed mouse mIgG in the version of mIgG-Tail, mIgG-Tailless, mIgG-Linker25-Tail or mIgG-Tail-K/2A. Also given is the expressing level of the endogenous IgM-BCR in these B cell sublines.

(b). Ca^{2+} mobilization analysis by flow cytometry in Ramos B cells expressing mIgG-Tail, mIgG-Tailless or mIgG-Linker25. Arrow indicates time point of the anti-mouse IgG (α-γ) stimulation. Representative data from three independent experiments is shown.

(**c**). The comparison of the Ca^{2+} mobilization response in Ramos B cells expressing mIgG-Tail or mIgG-Linker25-Tail upon the stimulation with α-γat very low concentration of 1 or 2 μg ml^{-1}.

Supplementary Fig. 3:

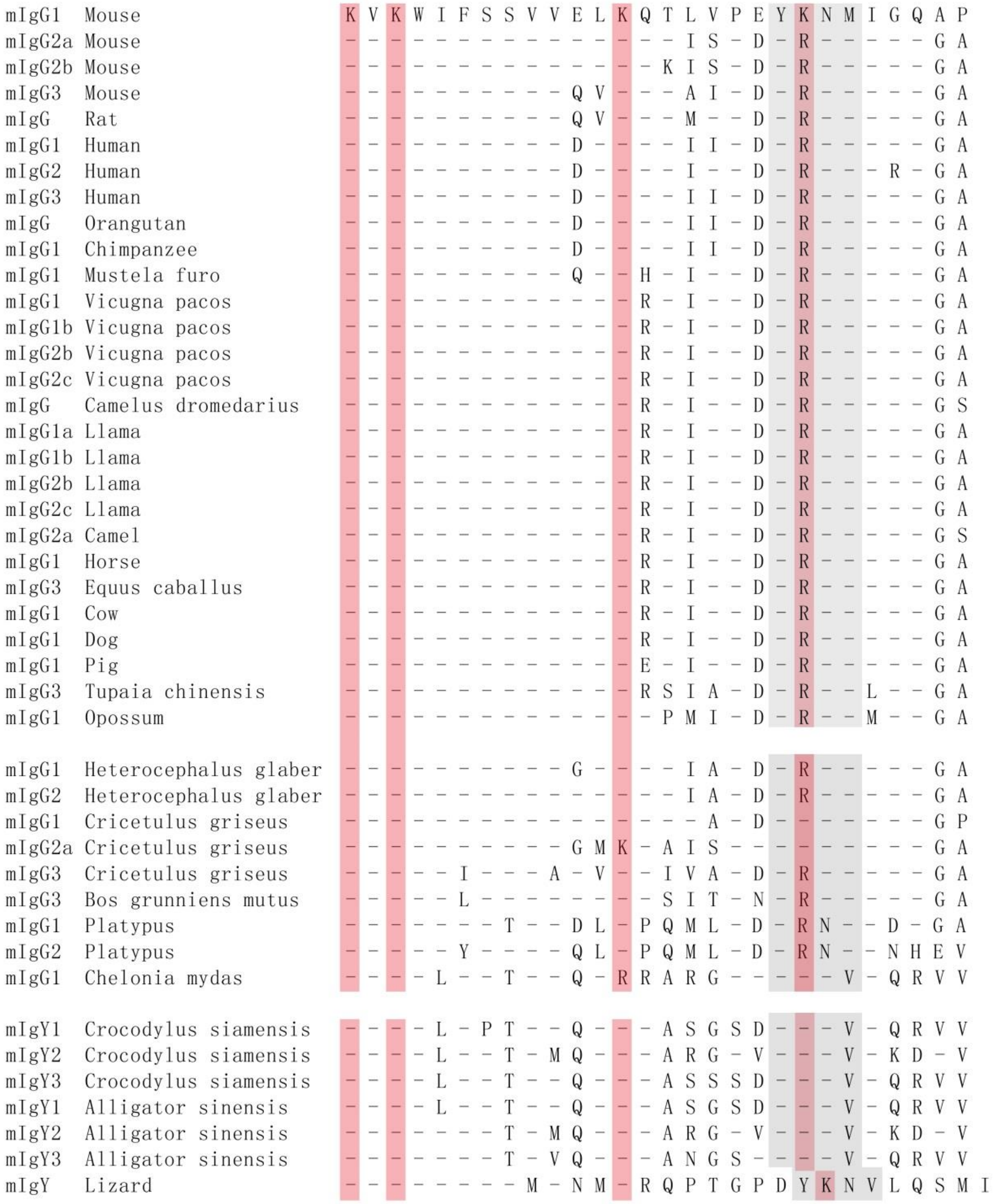

Supplementary Fig. 3: mIgG-Tail is evolutionary conserved

Alignments of the mIg-Tail aa sequences from several species. The ITT motifs (gray) and conserved basic residues (red) are highlighted.

Supplementary Fig. 4:

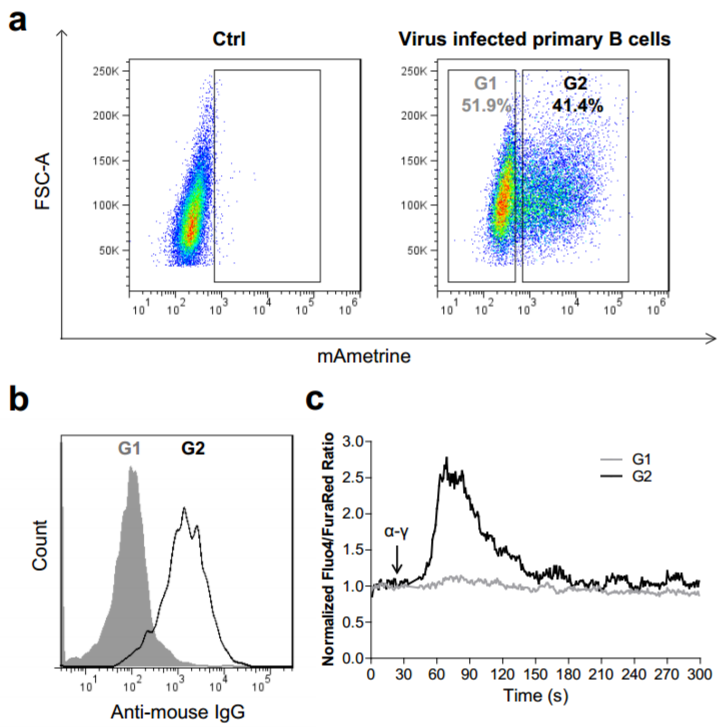

Supplementary Fig. 4: Primary B cells retrovirally expressing mIgG showed robust Ca^{2+} mobilization upon the crosslinking of IgG-BCR

(a). Mouse primary B cells retrovirally expressing mIgG and fluorescent protein mAmetrine by a standard pMSCV based retrovirus system with dual-promoter. Left panel showed the negative control without virus infection, and the right panel indicated that more than 40% of the infected primary B cells are mAmetrine positive.

(b, c). Only the mAmetrine positive primary B cells (G2) expressing IgG-BCR on the cell surface **(b)** showed the robust Ca^{2+} mobilization upon the activation by anti-mouse IgG (α-γ), while the negative control (G1) did not **(c)**.

Supplementary Fig. 5:

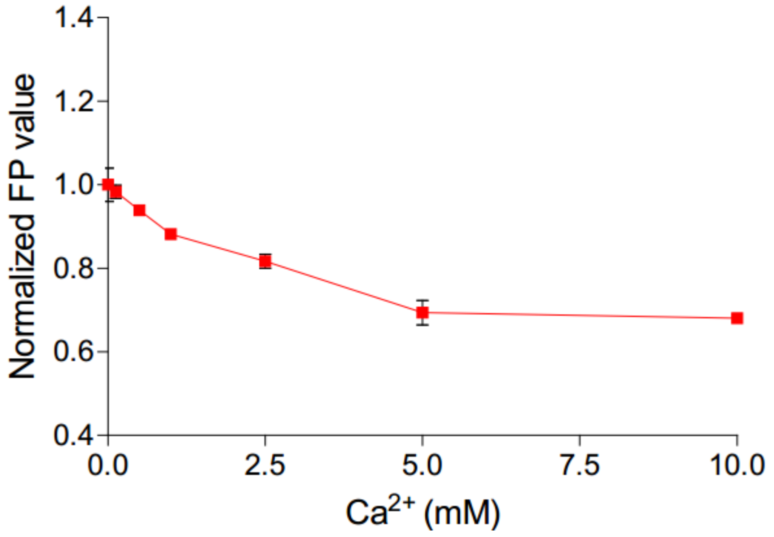

Supplementary Fig. 5: Ca^{2+} disrupt the binding between mIgG-Tail and the acidic lipid

The effect of Ca^{2+} on the interaction between CP488-mIgG-Tail peptide and acidic lipid POPG bicelles was assessed by FP assay. Ca^{2+} at indicated concentration were added to the peptide-lipid bicelle interaction system (100 nM CP488-mIgG-Tail peptide and 2.5 mM POPG bicelles), FP value was measured at each concentration with three repeat. FP value at different concentration was normalized to the FP value at the condition of no Ca^{2+} addition. Bars represent mean ± s.d. from three repeated experiments.
